# Inter-Individual Heterogeneity in Aerobic Training Adaptations: Systematic Review of the Evidence Base for Personalized Exercise Prescription

**DOI:** 10.3390/life15121932

**Published:** 2025-12-17

**Authors:** Haili Xiao, Jianchang Ren

**Affiliations:** 1 Institute of Sport and Health, Lingnan Normal University, Zhanjiang 524037, China; xiaohl@lingnan.edu.cn; 2 Guangdong Provincial Kay Laboratory of Development and Education for Special Needs Child, Lingnan Normal University, Zhanjiang 524037, China

**Keywords:** personalized exercise prescription, aerobic training adaptations, interindividual variability, trainability

## Abstract

Personalized exercise prescriptions require identifying inter-individual differences in exercise intervention effects. This review examined whether true inter-individual differences exist after accounting for measurement error. PubMed, Web of Science, and EMBASE databases were systematically searched. Inclusion criteria included: (1) original randomized controlled trials or controlled trials using standardized, supervised aerobic exercise interventions, or meta-analyses examining inter-individual differences in aerobic training adaptations; (2) explicitly identifying the assessment of inter-individual response variability as a research objective; (3) Reporting sufficient statistical information to quantify inter-individual variability. The search yielded a total of 3203 studies, and ultimately 78 studies were included for systematic review. Main findings included: (1) Few studies appropriately considered random measurement error and within-individual variability when quantifying inter-individual differences. (2) Analysis of both primary studies and existing meta-analyses revealed a consistent lack of statistical evidence for true inter-individual differences. (3) Observed inter-individual variability was primarily attributed to uncontrolled measurement error or within-individual variability, not true individual response differences. The evidence analyzed in this systematic review indicates that there is insufficient support for true inter-individual differences in the effects of aerobic exercise training intervention. Given physiological complexity and methodological limitations, any conclusions on exercise intervention heterogeneity or individual responsiveness require caution.

## 1. Introduction

“Exercise is medicine.” As a safe and effective intervention, exercise is increasingly central to disease prevention and management [1,2]. While general health benefits are well-established, the focus of exercise science has shifted toward personalized prescriptions to maximize individual outcomes [3,4]. This shift is motivated by the widely varying responses observed in training studies: even under identical training protocols, individuals exhibit a vast spectrum of phenotypic adaptations—ranging from “non-responders” to high responders—across various cardiorespiratory and metabolic parameters [5,6,7,8]. This spectrum of adaptations should be interpreted with caution, as measurement reliability can impact the assessment of individual responses. Additionally, random errors arising from inconsistent testing conditions or equipment variability may lead to the misclassification of individuals, potentially obscuring true physiological adaptations. Crucially, a key distinction must be made between “responder-by-protocol” effects and genuine heterogeneity in exercise response. Are the observed variations simply due to differing responses to a specific, standardized protocol, or do they reflect fundamental, underlying differences in how individuals adapt to exercise in general?

These observations appear to provide a compelling rationale for personalized exercise prescriptions. However, a critical methodological controversy complicates this translation: does this observed variability reflect true differential responses to exercise, or is it merely a statistical artifact? Biostatisticians emphasize that observed variability aggregates multiple sources, including random measurement error, daily biological fluctuation, and within-individual variability, alongside any true training effects [9,10,11]. A statistical artifact may arise from factors such as regression to the mean, technical error, or biological noise. Without a non-exercise control group to partition these variance components, non-training factors may be misidentified as physiological differences in responsiveness.

Supporting this methodological concern, recent meta-analyses challenge the prevalence of true inter-individual differences in aerobic training. For instance, analyses of VO_2max_ and endothelial function improvements indicate that observed variability is largely attributable to measurement error and biological noise rather than distinct training responses [12,13]. These findings contrast sharply with traditional interpretations, sparking debate on the validity of current personalized prescription strategies.

Clarifying this controversy is vital. If observed variations are primarily noise, current predictive biomarkers lack reliability. Conversely, if true differences exist after controlling for bias (in the magnitude of response), their determinants must be rigorously identified. Therefore, this review aims to: (1) systematically examine whether studies claiming inter-individual differences in aerobic training have rigorously partitioned random error from true response variability; and (2) based on methodological quality assessment, synthesize existing research evidence on inter-individual response differences in aerobic training adaptations to provide an objective evaluation of the scientific foundation for personalized exercise prescriptions.

## 2. Materials and Methods

This systematic review followed the Preferred Reporting Items for Systematic Reviews and Meta-Analyses (PRISMA) [14] checklist (for details on the review and PRISMA, see the Supplementary Materials Table S1). This project has been pre-registered on the Open Science Framework (OSF). (https://doi.org/10.17605/OSF.IO/E7NVD, 16 September 2025, Post-registration)

### 2.1. Eligibility Criteria

Studies included in the systematic review had to meet all of the following inclusion criteria: (1) Study type: (a) Original RCTs or controlled trials, (b) Systematic reviews with meta-analysis examining inter-individual differences in aerobic training adaptations. (2) Participants: human subjects. (3) Intervention characteristics: (a) For primary studies: standardized and supervised aerobic exercise training for at least 2 weeks, (b) For meta-analyses: pooled analyses of aerobic training interventions examining inter-individual variability in training responses. (4) Assessment of inter-individual response variability: studies explicitly investigated inter-individual differences in responses to aerobic training as a research objective and reported individual-level outcome data or sufficient statistical information to quantify inter-individual variability. It should be acknowledged that the exclusion of studies without explicit heterogeneity analysis introduces a potential selection bias. This limitation has been considered to ensure the focus remains on studies directly addressing inter-individual variability in aerobic training adaptations. Exclusion criteria (any one of the following led to exclusion): (1) Intervention characteristics: Unsupervised or non-standardized aerobic training protocols; Studies involving combined exercise modalities (aerobic with resistance training) or concurrent nutritional/pharmacological interventions, which were excluded to maintain methodological purity and avoid the confounding effects of different stimuli; Because the study focused on isolating the effects of standardized aerobic training, secondary aerobic outcomes reported in studies with combined aerobic exercise as the primary intervention were not considered; Training duration less than 2 weeks (for primary studies); (2) Publication type: Narrative reviews without quantitative synthesis; Conference abstracts or proceedings; Editorials, commentaries, letters; Case reports or case series. (3) Language: Non-English publications. (4) Accessibility: Full text unavailable through institutional libraries or publicly accessible online resources. For methodological rigor, the search was confined to peer-reviewed publications, thereby excluding grey literature which lacks a formal validation process. The scope was further restricted to English-language studies, as most high-impact international research is published in English, and due to practical constraints on translation. The authors acknowledge this approach may introduce a potential language bias, and consequently, a geographic bias.

### 2.2. Literature Search and Study Selection

We conducted a literature search in Web of Science, PubMed, and EMBASE on 1 September 2025. To ensure the comprehensiveness of the literature, we also reviewed the references of relevant studies to identify potential articles that were not listed in the main databases. The literature search covered the period from 1 January 2015, to 1 September 2025, because Atkinson and Batterham [10] first described in the exercise science literature in 2015 how to statistically estimate whether inter-individual differences in trainability exist, and outlined the standard deviation of individual response (SD_IR_) method [15]. Prior to 2015, standardized methods for quantifying inter-individual variability in response to exercise training were less common in the exercise science literature. Search terms primarily include ((“Exercise”[Mesh] OR “Aerobic exercise”[Title/Abstract] OR “Endurance training”[Title/Abstract] OR “Cardiorespiratory fitness”[Title/Abstract] OR “High-Intensity Interval Training”[Title/Abstract] OR “HIIT”[Title/Abstract]) AND (“Interindividual response”[Title/Abstract] OR “Individual variability”[Title/Abstract] OR “Response heterogeneity”[Title/Abstract] OR “Responder”[Title/Abstract]). A complete list of synonyms and related terms used in the search strategy is provided in Supplementary Materials Table S2. Titles and abstracts were extracted from the database searches, and duplicates were automatically removed using NoteExpress 4.2.0.10271 software. Literature screening and inclusion were performed independently by two authors, followed by consolidation of their screening results. All screening results were validated through mutual verification by the two reviewers, and cases that did not reach agreement were resolved through discussion until consensus was achieved.

### 2.3. Data Extraction

We used an Excel spreadsheet to extract the data, referencing the research data template proposed by Bonafiglia et al. [15]. We extracted basic study characteristics from all included studies (detailed in the Supplementary Materials Table S3). We extracted the change values and baseline values of the standard deviation for the exercise group and control group, which were used to calculate the SD_IR_ values. Additionally, for meta-analytic articles reporting SD_IR_ values for interindividual differences in aerobic training, we extracted the effect sizes of the SD_IR_ values along with their 95% confidence intervals. Missing standard deviation (SD) values were not imputed, ensuring the accuracy of the extracted data.

### 2.4. Risk of Bias Assessment

The risk of bias for each study was assessed using the Cochrane Collaboration Risk of Bias Assessment Tool, which categorizes the risk into three levels. A High Risk indicates that there is a clear risk of bias in one or more domains of the study, potentially affecting the validity of the results. In contrast, a Low Risk suggests that there is no evident risk of bias across the various domains, meaning the validity of the results remains intact. Finally, an Unclear Risk refers to situations where there is insufficient information available to make a definitive judgment about the risk of bias in a specific domain. These classifications provide a clear framework for evaluating potential bias and ensuring a rigorous assessment of study quality [16] (see Supplementary Materials Table S4).

### 2.5. Data Analysis

We adopted a three-tiered analytical strategy for a comprehensive and multi-layered assessment of the evidence for inter-individual differences in exercise response. The first tier established a foundational layer of evidence by extracting or calculating the inter-individual response ESD_IR_ from primary studies. For the second tier, we synthesized the reported response variance (SD_IR_^2^) from previously published meta-analyses to compare the consensus from our included studies with that of the wider field. The final tier of our analysis was a focused meta-analysis directly comparing the change in response variability (ΔSD) between exercise and non-exercise control groups. This approach isolates training-induced variability from background noise (measurement error, biological fluctuations). We selected systolic blood pressure (SBP) for this definitive test because its frequent reporting across studies provided the robust dataset necessary for such a comparison. This sequential method enabled us to first survey the evidence broadly, then critically test our core hypothesis using the most appropriate and controlled data. Moreover, we chose SBP over VO_2max_, which is often regarded as the primary outcome in this field, because the included literature showed that the exercise and non-exercise groups had the most comprehensive data on the change in standard deviation (ΔSD) for SBP. In contrast, the available data for VO_2max_ contained several missing values related to ΔSD.

#### 2.5.1. Quantifying Inter-Individual Differences in Training Response

The central challenge in determining whether true inter-individual differences exist is to separate exercise-specific variability from other sources of variation (measurement error, daily biological fluctuation, and behavioral changes). To achieve this, we calculated the standard deviation of individual response (SD_IR_), which quantifies the additional variability in the exercise group beyond that observed in the control group [9,10,13,17]:

(1) $${SD}_{IR}=\sqrt{{SD}_{EX}^{2}-{SD}_{CTRL}^{2}}$$

where SD_EX_ and SD_CTRL_ represent the standard deviations of change scores in the exercise and control groups, respectively. If SD_IR_ is significantly greater than zero, this indicates that exercise training introduces variability beyond measurement error, suggesting true inter-individual differences in adaptation. Conversely, if the 95% confidence interval (CI) for SD_IR_ includes or falls below zero, there is insufficient evidence for such differences. One fundamental assumption of the SDIR method is that within-individual variation (comprising measurement error and biological fluctuations) is equal between the intervention and control groups.

To enable comparison across studies with different measurement scales, we standardized SD_IR_ by dividing it by the pooled baseline standard deviation, yielding the effect size ESD_IR_ [9,18]:

(2) $${ESD}_{IR}=\frac{{SD}_{IR}}{{SD}_{baselinePooled}}$$

SD_baselinepooled_ refers to the pooled standard deviation at baseline. Using baseline SD helps to minimize bias, as it focuses solely on the baseline measurements of each subject and maintains the integrity of individual data. This approach reduces the risk of conflating natural variations with those induced by the intervention, leading to more reliable and unbiased results in the evaluation of the intervention’s effects. If the 95% confidence interval for SD_IR_ is above zero, it indicates inter-individual differences in aerobic training adaptation. If the confidence interval crosses zero or is negative, it suggests no inter-individual differences in aerobic training adaptation. Standard errors for SD_IR_ estimates and 95% confidence limits were calculated using established variance estimation methods. Detailed formulas for standard error calculations and CI construction are provided in Appendix A.

#### 2.5.2. Meta-Analytic Synthesis

Given the expected heterogeneity in training protocols and participant characteristics, we employed an inverse variance heterogeneity (IVhet) model rather than traditional random-effects models. Random-effects models can be unreliable when there is a lot of inconsistency, potentially leading to false confidence in the results. The IVhet model is designed to handle this kind of variation, giving us more trustworthy estimates of the true effect [19]. We did not pool studies with substantial heterogeneity in training protocols or participant characteristics in the primary meta-analysis, prioritizing internal validity and accurate effect estimation over broader generalizability. Analyses were performed using Microsoft Excel and Meta XL (version 5.3), with all relevant statistical tests being two-tailed.

## 3. Results

### 3.1. Study Selection

**Figure 1:**
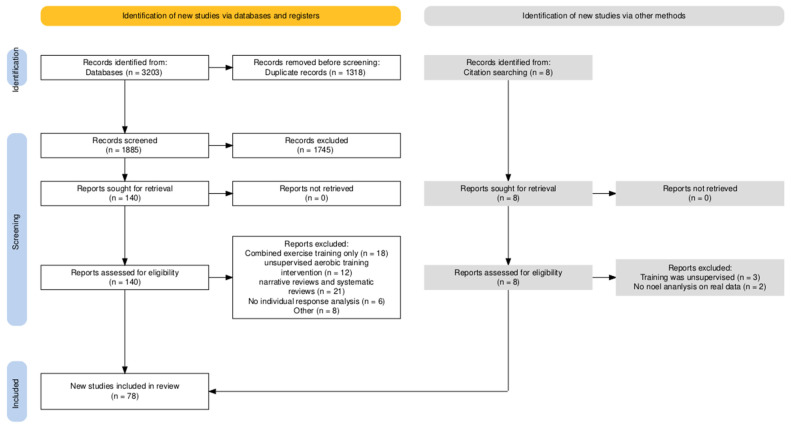
Flow diagram of the study selection process.

The literature search yielded 3203 records. Using NoteExpress software, 1318 duplicate records were removed. A total of 1885 articles proceeded to the title and abstract screening phase. After reviewing the abstracts, 1745 irrelevant articles were excluded. Full texts of 140 articles were downloaded. After reading the full texts, 65 were excluded (reasons for exclusion include: 18 reports of combined exercise training only, 12 reports of unsupervised aerobic training intervention, 21 narrative and systematic reviews, 6 reports with no individual response analysis, and 8 for other reasons), leaving 75 articles. Three additional articles meeting the inclusion criteria were identified through reference tracing. The systematic review ultimately included 78 articles, including 8 meta-analyses. Detailed information on the included studies is provided in Supplementary Materials Tables S3 and S6. Figure 1 illustrates the literature screening process.

### 3.2. Methods for Quantifying Aerobic Exercise Adaptation Heterogeneity and Classification of Individual Responses

Only 15 studies (Leifer et al. [20], n = 1188; Williamson et al. [21], n = 180; Hecksteden et al. [18], n = 36; Bonafiglia et al. [22], n = 109; Hammond et al. [23], n = 181; Walsh et al. [24], n = 143; Yu et al. [25], n = 78; Mattioni er al. [26], n = 58; Lea et al. [27], n = 131; Bonafiglia et al. [28], n = 29; Domaradzki et al. [29], n = 141; Domaradzki et al. [30], n = 73; Cano-Montoya et al. [31], n = 39; Moreno-Cabañas et al. [32], n = 264; Metcalfe and Vollaard [33], n = 117) employed statistical methods to estimate random measurement error or within-subject variability. Supplementary Table S3 provides additional information on the classification approaches.

Detailed information on the statistical methods used to estimate random variation and within-individual variation in the included studies is presented in Table 1. Although TE_M_ estimates random variation, it does not account for within-individual variation. The cross-over trial design is methodologically robust. Through within-subject comparisons and multiple repetitions, it enables the model to distinguish and estimate “random measurement error,” “intra-subject variability,” and “subject × training interaction.” However, this requires a crossover trial with multiple intervention and control periods, each separated by adequate washout periods. Furthermore, the carryover effects of the initial training intervention remain unclear, making it difficult to precisely determine the necessary washout periods between phases. The premise of the SD_IR_ method is that the combined effect of “random variation plus within-subject variation” is equal in the intervention and control groups. However, even with random assignment, if within-subject variation cannot be estimated in the exercise and control groups, the effects may differ.

### 3.3. Risk of Bias Assessment

The risk of bias assessment reveals methodological weaknesses that warrant a cautious interpretation of our findings (Supplementary Materials Table S4). Performance bias and reporting bias were the predominant concerns, with 68.6% and 50.0% of studies rated as high risk, respectively. Detection bias was predominantly unclear (64.3%) due to insufficient reporting of outcome assessor blinding, while attrition bias was generally low risk (74.3%). Randomization and allocation concealment quality was moderate, with approximately one-quarter to one-third of studies showing unclear or high risk.

These biases have specific implications for the interpretation of SD_IR_ estimates and the existence of inter-individual differences. Performance bias (high risk in 68.6% of studies) arises from participants’ awareness of group allocation, which may systematically inflate the variance in exercise groups through differential adherence, motivation, or placebo effects. Additionally, many of the assessed outcomes are dependent on the effort exerted by participants, which can further complicate the bias. Crucially, if such non-physiological factors increase SD_EX_ more than SD_CTRL_, the calculated SD_IR_ would spuriously suggest inter-individual differences even when true training-specific variability is minimal. This directional bias undermines confidence in positive SD_IR_ findings.

Detection bias (unclear risk in 64.3% of studies) compounds this concern. Unblinded outcome assessors may unconsciously record more favorable or variable results in exercise groups, artificially elevating SD_EX_. When combined with performance bias, this creates a systematic tendency to overestimate SD_IR_, particularly in subjective or effort-dependent outcomes (e.g., exercise capacity tests). Therefore, studies lacking double-blinding are at heightened risk of false-positive conclusions regarding inter-individual variability.

Reporting bias (high risk in 50.0% of studies) introduces a different threat: publication and selective outcome reporting favor studies showing significant heterogeneity or “responder” subgroups. This creates an ascertainment bias in the available literature, where studies finding no evidence of inter-individual differences (SD_IR_ ≈ 0 or negative) are systematically underrepresented. Consequently, our synthesis may overestimate both the prevalence and magnitude of true inter-individual differences across the field.

Given these converging biases, we emphasize key limitations in interpreting our findings: (1) Positive SD_IR_ estimates should be interpreted with caution, as performance and detection biases likely inflate variance estimates in exercise groups; (2) The proportion of studies demonstrating significant inter-individual differences may be overestimated due to publication bias against null findings; (3) Studies with rigorous blinding and control group designs warrant greater evidential weight in adjudicating the existence of true inter-individual variability. (4) In most cases, the variance inflation factor (VIF) between 1.5 and 2.0 may be effective. This means that if a true effect exists in the study but has not been detected due to insufficient sample size, moderate variance inflation may increase the chances of detecting inter-individual differences [34,35]. While the overall pattern of findings provides preliminary evidence, definitive conclusions require replication in bias-minimized designs.

### 3.4. Studies Estimating Interindividual Differences in Response to Aerobic Training

**Figure 2:**
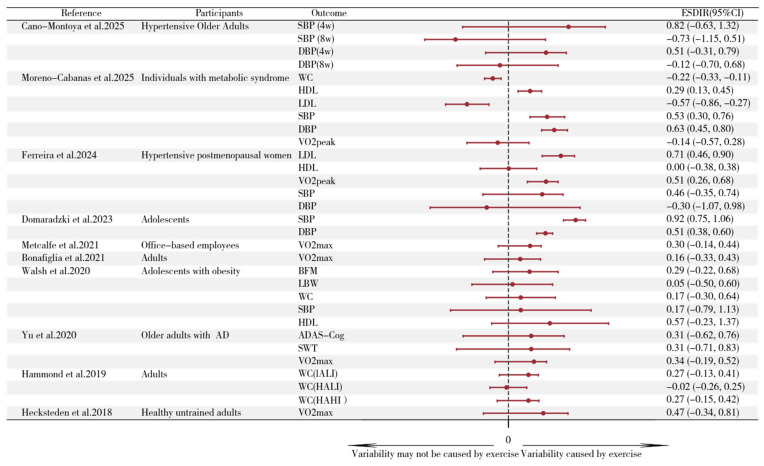
Forest plot of ESDIR estimates with 95% CIs across studies. SBP, systolic blood pressure; DBP, diastolic blood pressure; VO_2max_, maximal oxygen consumption; HDL-c, high-density lipoprotein-cholesterol; LDL-c, low-density lipoprotein-cholesterol; VO_2peak_, peak oxygen uptake; BFM, Body Fat Mass; LBM, Lean Body Mass; WC, Waist Circumference; ADAS-Cog, AD Assessment Scale-Cognition; SWT, shuttle walk test. CI: confidence interval. This figure displays ESD_IR_ estimates from 31 exercise-control comparisons across 10 studies, quantifying the magnitude of inter-individual variability in training responses after accounting for measurement error and natural biological fluctuation. Each data point represents the standardized additional variability observed in the exercise group relative to the control group for a specific outcome measure. ESD_IR_ > 0 with 95% CI entirely above zero (solid symbols above the dashed line): Provides evidence for true inter-individual differences in training response. These cases suggest that individuals respond heterogeneously to the same training program for that specific outcome, potentially warranting personalized prescription strategies. 95% CI crossing zero (symbols spanning the dashed line): Indicates insufficient evidence to distinguish training-induced variability from random noise. The observed differences among individuals may primarily reflect measurement error or day-to-day biological variation rather than true differential training responses. ESD_IR_ ≤ 0 or 95% CI below zero (symbols at or below the dashed line): Suggests no detectable inter-individual differences in response, or that the control group exhibited greater variability. In these cases, the evidence does not support tailoring interventions based on expected individual variability for that outcome. Cano-Montoya et al., 2025 [31], Moreno-Cabanas et al. 2025 [32], Ferreira et al. 2024 [36], Domaradzki et al. 2023 [29], Metcalfe and Vollaard, 2021 [33], Bonafiglia et al., 2021 [28], Walsh et al., 2020 [24], Yu et al., 2020 [25], Hammond et al., 2019 [23]; Hecksteden et al., 2018 [18].

**Figure 3:**
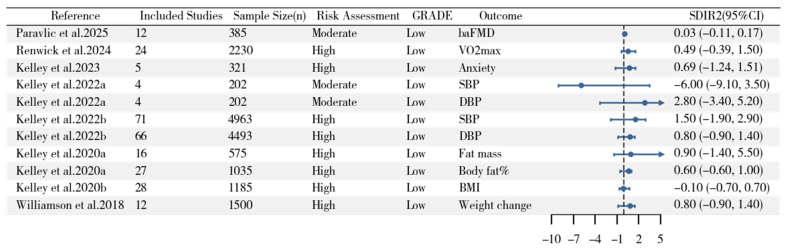
Forest Plot of SD_IR_^2^ Estimates and Their 95% Confidence Intervals from Meta-Analyses of Inter-Individual Differences in Adaptations to Aerobic Training. _ba_FMD, Brachial artery flow-mediated dilatation; VO_2max_, maximal oxygen consumption; SBP, systolic blood pressure; DBP, diastolic blood pressure; BMI, body mass index. CI: confidence interval. This figure synthesizes findings from 11 previously published meta-analyses examining inter-individual variability in response to aerobic training across diverse outcomes. Each data point represents the pooled estimate of response variance (SD_IR_^2^) from a separate meta-analysis, quantifying whether exercise training introduces variability beyond that observed in control conditions. SD_IR_^2^ > 0 with 95% CI entirely above zero: Would indicate meta-analytic evidence for true inter-individual differences in training response at the population level, suggesting systematic heterogeneity in how individuals adapt to aerobic exercise for that outcome. 95% CI crossing zero (all cases in this figure): Indicates that the meta-analytic evidence does not support the existence of true inter-individual differences. Wider confidence intervals may reflect small sample sizes. The observed variability in exercise groups is statistically indistinguishable from measurement error and biological noise captured by control groups. This pattern suggests that apparent “responders” and “non-responders” in individual studies may largely reflect random variation rather than stable individual characteristics. SD_IR_^2^ ≤ 0 or negative values: Would suggests that the control group showed greater variability than the exercise group—a counterintuitive finding that typically indicates data quality issues rather than a biological phenomenon. Possible explanations include inconsistent measurement conditions in the control group, or chance fluctuations due to small sample sizes. Such results should be interpreted with caution and prompt re-examination of study methodology. All 11 meta-analytic estimates from eight independent meta-analyses had 95% CIs that included zero, spanning outcomes including vascular function (baFMD), cardiorespiratory fitness (VO_2_max), blood pressure (SBP, DBP), and body composition (BMI). This consistent pattern across diverse outcomes and research groups strengthens the conclusion that true inter-individual differences in aerobic training response may be less prevalent than traditionally assumed. The absence of meta-analytic evidence for inter-individual differences across multiple outcomes has important implications for exercise prescription practice. It challenges the validity of “exercise responder” classification systems that assign individuals to categories (e.g., “high responder,” “low responder,” “non-responder”) based on single measurements without accounting for measurement error. Such classifications may be unreliable if individuals do not exhibit stable differential responses. These findings suggest that the focus of personalized exercise prescription should shift from attempting to predict individual variability in standard outcomes (e.g., VO_2_max, blood pressure) toward identifying modifiable factors that improve average responses for all individuals (e.g., optimizing training dose, adherence strategies, behavioral support).Practitioners should interpret claims about “personalized exercise genomics” or “precision exercise medicine” critically, particularly when such approaches promise to predict individual training responses based on baseline characteristics, as the underlying assumption of substantial true inter-individual variability is not strongly supported by controlled studies. Paravlic et al. 2025 [12], Renwick et al. 2024 [11], Kelley et al. 2023 [37], Kelley et al. 2022a [38], Kelley et al. 2022b [39], Kelley et al. 2020a [40], Kelley et al. 2020b [41], Williamson et al. 2018 [42].

We calculated ESD_IR_ values and their 95% confidence intervals (CIs) across 10 studies, comprising 31 groups of data as presented in Figure 2. Detailed information regarding participant characteristics and aerobic intervention protocols for each study is provided in Supplementary Materials Table S5. To minimize heterogeneity and more accurately represent the original data, we did not pool the data. Given the substantial heterogeneity in participant characteristics and intervention protocols across studies, we did not calculate an overall pooled effect size. However, the studies presented mixed evidence, with the majority of ESD_IR_ 95% CIs either crossing zero or lying below zero. The ESD_IR_ estimates from Moreno-Cabanas et al. (2025), (n = 264) [32] revealed that the 95% CIs for HDL, SBP, and DBP lay above zero. The ESD_IR_ estimates from Ferreira et al. (2024), (n = 65) [36] demonstrated that the 95% CIs for LDL and VO_2peak_ lay above zero. The ESD_IR_ estimates from Domaradzki et al. (2023), (n = 141) [29] showed that the 95% CIs for SBP and DBP lay above zero. For all remaining studies (Cano-Montoya et al., 2025 [31], n = 26; Metcalfe and Vollaard, 2021 [33], n = 25; Bonafiglia et al., 2021, n = 29 [28]; Walsh et al., 2020 [24], n = 91; Yu et al., 2020 [25], n = 26; Hammond et al., 2019, n = 97 [23]; Hecksteden et al., 2018 [18], n = 36), the 95% CIs for ESD_IR_ estimates either crossed zero or lay below zero. Additionally, the forest plot in Figure 3 displays 11 SD_IR_^2^ estimates and their 95% confidence intervals (CIs) from meta-analyses of different indicators of inter-individual variability in adaptations to aerobic training. These 11 datasets were derived from eight meta-analyses[11,12,37,38,39,40,41,42]. The 95% CIs for SD_IR_^2^ across all outcome measures include zero. Detailed information for each meta-analysis is provided in Supplementary Materials Table S6. Of the 31 ESD_IR_ estimates from primary studies, 24 (77%) had 95% CIs that crossed or were below zero (Figure 2). Similarly, all 11 SD_IR_^2^ estimates from previous meta-analyses had 95% CIs that included zero (Figure 3), indicating a consistent lack of evidence for true inter-individual differences across the literature. (When ${SD}_{EX}^{2}-{SD}_{CTRL}^{2}$ is negative, based on statisticians’ recommendations, the sign should be changed first, and the result should be presented as a negative standard deviation, interpreted as the variability in the control group being greater than that in the experimental group.)

### 3.5. Separate Group Meta-Analysis on Standard Deviation of Change Scores

Our initial analysis of primary studies and existing meta-analyses revealed a consistent lack of statistical support for significant inter-individual differences in training response for most outcomes (Figure 2 and Figure 3). To investigate this pattern more deeply, we conducted a focused meta-analysis on a single, representative outcome. Systolic blood pressure (SBP) was selected for this detailed analysis because it was one of the most frequently reported outcomes, providing a robust dataset.

**Figure 4:**
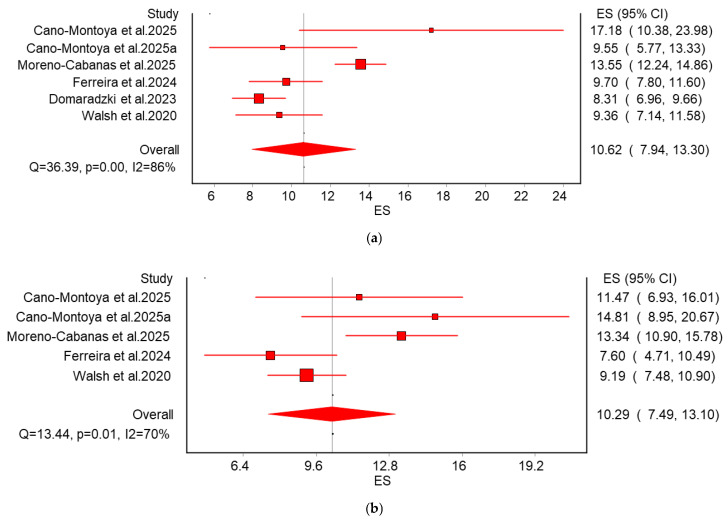
Standard deviation of change scores across excise groups (**a**) and control groups (**b**). Meta-analysis showed that the estimated standard deviation of change was similar between the exercise group (**a**) (10.64 [95% CI 7.94–1.30]) and the control group (**b**) (10.92 [95% CI 7.49–13.10]), suggesting that most of the deviation in change scores in exercise interventions primarily arises from measurement error. The thin central line represents the mean change score for the intervention. The standard deviation of change for each study, together with its 95% confidence intervals, is shown on the right side of each panel. CI: confidence interval. The similar pooled estimates for change in standard deviation (ΔSD) and overlapping confidence intervals indicate that aerobic training did not systematically increase inter-individual variability in systolic blood pressure. This suggests that the observed variation in individual responses is statistically indistinguishable from the measurement error and biological fluctuation captured in the control group, rather than reflecting true physiological heterogeneity. These findings challenge the validity of classifying individuals as “responders” or “non-responders” based on single-assessment changes. Since true inter-individual differences appear negligible, the data support the use of standardized, evidence-based exercise prescriptions for blood pressure management, as there is insufficient evidence to justify personalizing training dosages based on anticipated response variability. Cano-Montoya et al. 2025 [31], Moreno-Cabanas et al. 2025 [32], Ferreira et al. 2024 [36], Walsh et al. 2020 [24].

For this analysis, we used the difference in post- and pre-training standard deviation (ΔSD = SD_post_ − SD_pre_) as the effect size for both exercise and control groups, drawing from studies listed in Figure 2 (see Formula (1)) [24,29,32,36]. The results are presented in Figure 4. Figure 4a shows the pooled ΔSD for the exercise group as 10.62 (95% CI 7.94–13.30), (Q = 36.39, I^2^ = 86) while Figure 4b shows the pooled ΔSD for the control group as 10.29 (95% CI 7.49–13.10), (Q = 13.14, I^2^ = 70). The ΔSD estimates were similar between groups. Based on the standard deviation of the change score, the SEM for the control group was 1.78 (0.8–2.9), and for the exercise group, it was 1.48 (0.67–3.5). The current evidence fails to provide compelling support for additional between-subject variability in SBP response to aerobic training, as no statistically significant increase in variability was found relative to control groups. The similar ΔSD in exercise and control groups suggests the observed variability is not training-specific. The ΔSD of approximately 10 mmHg in the exercise group and the control group exceeds the error range for a single blood pressure measurement (±3 to ±5 mmHg). Generally, a change in systolic blood pressure greater than 5 mmHg is considered to have potential clinical significance. Given the differences in the number of study participants, their ages, demographics, and physical conditions, this variability may be attributed to natural fluctuations, daily activities, psychological factors, or environmental changes.

### 3.6. Standard Deviation of Individual Response

**Figure 5:**
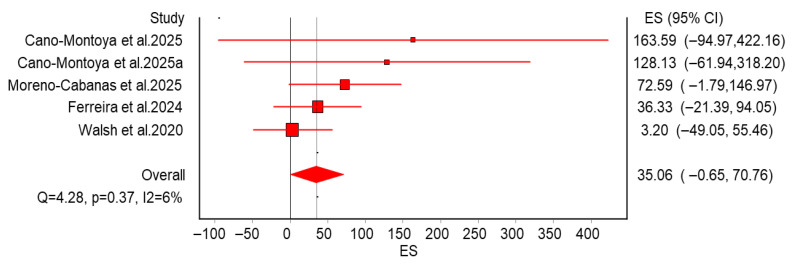
Meta-analysis of SD_IR_^2^ for systolic blood pressure response to aerobic training. The mean SD_IR_^2^ estimate (35.06 [95% CI −0.65 to 70.76]) fails to provide evidence that individual trainability exists for the SBP response. The SD_IR_^2^ estimates for individual studies, along with their 95% confidence intervals, are shown on the right side of the panel. CI: confidence interval. The 95% CI spans from a negative value (−0.65) to a positive value (70.76), crossing the line of null effect (zero). Statistically, this indicates insufficient evidence to conclude that true inter-individual differences exist. It suggests that the variability observed in the exercise groups is not significantly distinct from the random variation and measurement noise observed in non-exercising control groups. The inclusion of negative values in the lower limit suggests that in some instances, the control group exhibited equal or greater variability than the exercise group. This is indicative of measurement error or random sampling fluctuation dominating the data, rather than a true biological phenomenon of differential “trainability.” For applied exercise science, this finding challenges the utility of classifying patients as “high” or “low” responders for systolic blood pressure. Since the added variance from training is statistically negligible, standardized aerobic training guidelines are likely to be effective for the majority of individuals. Practitioners should prioritize adherence to proven standard protocols rather than attempting to tailor interventions based on an unproven assumption of high individual response heterogeneity. Cano-Montoya et al., 2025 [31], Moreno-Cabanas et al. 2025 [32], Ferreira et al. 2024 [36], Walsh et al., 2020 [24].

We calculated the SD_IR_^2^ for SBP in each study included in Figure 4 and conducted a meta-analysis based on this. Figure 5 presents the meta-analysis results: the pooled effect size was 35.06 (95% CI −0.65 to 70.76), (Q = 4.28, I^2^ = 6%). The results of the meta-analysis indicate a Q value of 4.28, suggesting that there is no significant heterogeneity among the studies included. The I^2^ value is 6%, indicating that only 6% of the total variability can be attributed to between-study differences, which reflects a high level of consistency among the studies. Since the 95% CI spans zero, current evidence is insufficient to indicate additional between-individual variability in SBP response to aerobic exercise interventions. Detailed information on the included studies is provided in Supplementary Table S5.

## 4. Discussion

The main findings of this review are as follows: (1) Among the included studies, only a small number (n = 15) considered random variation and within-subject variation when assessing inter-individual differences. (2) Analysis of both primary studies and existing meta-analyses revealed a consistent lack of statistical evidence for true inter-individual differences, as the 95% confidence intervals for response variability consistently included zero. (3) The observed inter-individual variability in aerobic exercise intervention effects within the existing literature may be attributable to random variation or intra-individual variation.

### 4.1. Strength of the Study and Summary of Findings

We synthesized results from 78 studies. The majority of included studies failed to consider random variation and within-subject variation when evaluating inter-individual differences. This suggests that the majority of existing studies lack sufficient evidence to interpret the observed variability in aerobic training adaptations as true individual differences in intervention effects. To support this inference, we calculated 31 ESD_IR_ values from selected studies, with most studies showing ESD_IR_ values either below zero or crossing zero (24/31) (Figure 2). Although the overall pattern was consistent, a few exceptions for specific outcomes (e.g., HDL, SBP, and DBP in Moreno-Cabanas et al. [32].) were observed. Additionally, we synthesized 11 outcome measures from 8 recent meta-analyses examining inter-individual differences in aerobic training adaptations. Across all measures, the 95% confidence intervals for SD_IR_^2^ effect sizes crossed zero. To disentangle random variation and within-subject variation from the observed inter-individual differences, a meta-analysis was conducted on the ΔSD of SBP for both exercise and control groups in selected studies listed in Figure 2 (see Formula (1)). After disentangling random variation and within-subject variation from the observed inter-individual differences, the synthesized results from the included studies provided no compelling evidence for the existence of inter-individual differences in exercise intervention effects.

### 4.2. Do No Inter-Individual Differences Exist in Exercise Intervention Effects?

To improve exercise interventions, researchers seek predictors of outcomes, noting that understanding molecular mechanisms behind individual variability could inform personalized prescriptions [43]. Some suggest that changes in mRNA expression after a single exercise session can predict long-term results [44]. However, evidence challenges this. Islam et al. found poor reproducibility in mRNA changes after two identical cycling tests, indicating high variability in acute responses among subjects [45]. Additionally, Dankel et al. demonstrated that observed inter-individual variability in exercise effects is mainly due to random variation rather than true individual differences, based on controlled training sessions with a non-intervention group [46]. Likewise, linking acute gene expression changes to long-term outcomes presents methodological challenges [47]. Our analysis using the standard deviation of individual response (SD_IR_) method revealed no statistically significant inter-individual differences in exercise effects after accounting for random and within-individual variability. This aligns with recent meta-analyses showing that variability in responses is largely attributable to non-specific factors like measurement error rather than real differences in adaptive capacity [12]. In conclusion, current evidence does not support true heterogeneity once measurement error is controlled.

A comparative perspective from pharmacological research provides useful context for interpreting these findings. Drug treatment effects demonstrate clear inter-individual differences; for example, pharmacological interventions for glycemic control exhibit significant inter-individual variability that primarily stems from genetic heterogeneity [48,49]. Pharmacogenomic studies indicate that genetic variants (such as single nucleotide polymorphisms) influence drug responses [50]; the glycemic response to metformin is heritable, with genetic variants in OCT2, CPA6, and PRPF31 closely associated with its efficacy and exhibiting inter-ethnic differences [51,52,53,54,55,56]. In contrast, exercise training involves complex multi-organ, multi-pathway networks (including energy metabolism, insulin signaling, cardiovascular adaptation, epigenetic modifications, etc.), often with redundant and compensatory mechanisms. Two scenarios may explain our findings: First, the multi-system nature of exercise may produce relatively uniform basic physiological adaptations across most individuals, with mechanisms sufficiently robust and redundant that true inter-individual differences in core adaptations (e.g., VO_2max_, insulin sensitivity) are minimal or absent. Second, genuine individual differences may exist but are masked by the complexity of exercise responses, measurement imprecision, or heterogeneous study populations.

While our analysis did not detect significant inter-individual differences using current methods, several considerations warrant cautious interpretation of the broader implications. First, the SD_IR_ method requires adequate statistical power and measurement precision, which may be limited in some included studies, potentially reducing sensitivity to detect small but genuine individual differences. Second, candidate gene studies suggest potential genetic modifiers of exercise response; for instance, some evidence indicates that elderly individuals carrying the ACE (D) allele may exhibit different tendencies in functional outcomes such as walking speed and physical performance batteries [57]. However, these findings are predominantly based on small-sample studies lacking validation through rigorous controlled designs and SDIR methodology. Such associations may reflect subgroup-specific effects, gene-environment interactions, or false positives rather than evidence for general inter-individual variability across the population. Third, most included studies examined aerobic capacity, cardiovascular function, and cardiometabolic outcomes; individual differences may be more pronounced in other adaptation domains not comprehensively assessed here, such as neuromuscular adaptations, pain tolerance, or cognitive responses. Fourth, our analysis focused on whether individuals differ in magnitude of response to standardized protocols; it does not address whether individuals might respond better to different types of exercise (e.g., HIIT vs. moderate continuous training), which represents a distinct question for personalized prescription. Fifth, our findings do not preclude the potential for a ‘responder-by-protocol’ interaction. That is, while individuals may not differ in their physiological response to a standardized protocol, they might show stronger adherence and achieve better long-term outcomes with a protocol tailored to their personal preferences. This suggests that a ‘response’ can also be behavioral, where personalization based on enjoyment and sustainability, rather than initial physiological markers, could be a key factor in optimizing health benefits.

Our findings challenge the current evidence base and theoretical foundation for precision exercise medicine in the domains examined, particularly for cardiorespiratory and basic metabolic adaptations to aerobic training. The widespread assumption that individuals exhibit large, stable, genetically determined differences in “trainability” for outcomes like VO_2max_ is not supported by rigorous analysis that accounts for measurement error and biological variability. This has important implications: biomarkers or genetic predictors proposed to identify “responders” and “non-responders” based on observational studies may lack validity if the response heterogeneity they attempt to explain is largely artifactual. Personalized exercise prescription strategies based on such predictors require re-evaluation.

However, these findings should not be interpreted as refuting the value of individualized exercise prescription more broadly. Exercise programming can and should be individualized based on factors such as baseline fitness, health status, injury history, preferences, and behavioral factors affecting adherence—even if the ultimate physiological adaptations do not vary substantially across individuals given adequate stimulus. Furthermore, our conclusions are limited to the specific outcomes and populations examined; definitive resolution of whether clinically meaningful individual differences in exercise trainability exist requires future research with larger samples enabling adequate statistical power, genetic stratification to test specific biological hypotheses, precise phenotyping with validated, low-error measurements, standardized training protocols with verified adherence, and examination of diverse outcomes across multiple physiological domains. Distinguishing between “observed variability” and “genuine individual response differences” through rigorous study design and statistical methods remains essential for advancing both exercise science and evidence-based practice.

### 4.3. Implications for Personalized Exercise Prescription

Our finding that significant inter-individual differences in aerobic training response are not detectable after accounting for measurement error and biological variation challenges foundational assumptions underlying “precision exercise medicine.” The current paradigm assumes that individuals exhibit substantial, stable heterogeneity in physiological responses to standardized training, justifying efforts to develop predictive algorithms (incorporating genetic markers, baseline characteristics, or phenotypic profiles) to identify “responders” versus “non-responders” a priori. However, if true inter-individual differences are minimal or absent—as our data suggest—attempts to stratify individuals based on predicted trainability become statistically untenable. An individual classified as a “non-responder” in one trial may exhibit favorable responses when retested under identical conditions, not due to physiological change but simply due to natural statistical fluctuation or imprecision in the measurement process. This undermines the validity of predictive tools and algorithms claiming to optimize training based on individual response profiles: without substantial true heterogeneity, such approaches cannot meaningfully outperform standardized, evidence-based protocols.

These findings necessitate redefining personalization in exercise prescription—shifting focus from predicting differential physiological responses to optimizing behavioral implementation of universal training principles. Fundamental principles such as progressive overload, specificity, and consistency apply broadly across individuals; thus, personalization should emphasize how these principles are delivered rather than whether they apply. The impactful individualization occurs at the behavioral and contextual level: tailoring exercise modality to personal preferences (e.g., cycling versus running), scheduling sessions to accommodate lifestyle constraints, framing goals around personally meaningful outcomes, and incorporating preferred social contexts (group versus individual training). A physiologically “suboptimal” program that is consistently followed invariably outperforms a theoretically “perfect” program abandoned due to inconvenience or misalignment with personal circumstances. Practitioners should therefore abandon unreliable response-based stratification, prioritize adherence-enhancing strategies (collaborative goal-setting, preference assessment, barrier identification), and use evidence-based guidelines as foundations, modifying primarily for adherence rather than predicted response.

Critically, the absence of stable between-individual heterogeneity does not preclude the importance of dynamic within-individual variation. Even if individuals possess similar average trainability, any given person’s readiness and responsiveness fluctuates substantially over time due to accumulated fatigue, stress, sleep quality, illness, and life circumstances. This temporal variability is actionable through responsive programming. The framework of within-individual optimization offers a conceptually superior alternative to between-individual prediction models, emphasizing dynamic load modulation based on real-time feedback rather than fixed prescriptions. Practitioners can adjust daily or weekly training loads using subjective markers (daily wellness questionnaires assessing recovery, mood, sleep quality, muscle soreness) and objective physiological indicators (heart rate variability, resting heart rate, performance-based metrics such as velocity-based training or heart rate recovery). Importantly, this approach does not require complex technology—simple strategies such as rating perceived recovery on a numerical scale can guide meaningful adjustments. This framework reduces injury and overtraining risk by scaling back during periods of inadequate recovery, maximizes training adaptations by increasing load when individuals are well-recovered, and enhances long-term adherence by actively involving individuals in the monitoring and decision-making process.

Our findings are consistent with the within-individual optimization theoretical framework, which distinguishes between mechanism-level universality and context-level variability. Specifically, while the physiological mechanisms underlying training adaptation (such as mitochondrial biogenesis, cardiac remodeling, and vascular adaptation) operate according to universal dose–response principles, the actual outcomes achieved by individuals in real-world settings are substantially influenced by behavioral and contextual factors including adherence patterns, daily lifestyle behaviors, timing of measurements, and psychosocial circumstances. This framework explains why controlled studies demonstrate minimal detectable inter-individual response differences while real-world outcomes appear heterogeneous—the variability observed in practice reflects differences in how well training principles are implemented and sustained, rather than inherent differences in physiological trainability.

### 4.4. Limitations and Implications

This review has several important limitations that should be considered when interpreting the findings. First, our search strategy specifically targeted studies explicitly examining inter-individual differences in exercise training responses. Consequently, we may have excluded relevant randomized controlled trials that collected individual-level outcome data but did not frame individual response variability as a primary research objective. This focused search approach, while necessary to identify studies employing appropriate analytical methods, may have limited the pool of potentially informative data. Second, the included studies exhibited substantial heterogeneity in sample characteristics, training protocols (duration, frequency, intensity, modality), outcome measurement methods, and statistical analysis strategies. This heterogeneity constrained our ability to conduct quantitative synthesis for certain outcomes and limited the precision of cross-study comparisons. The observed differences in training adaptation between individuals need to exclude measurement errors and biological variations. Therefore, our studies only included those with control groups, which may significantly impact the external validity of the results. Excluding studies without control groups may limit the generalizability of the conclusions, as these studies might provide information on long-term effects, rare events, and adverse reactions.

Third, a key assumption of the SD_IR_ method: that within-individual variation (comprising measurement error and biological fluctuations) is equal between the intervention and control groups. While this is a standard assumption in meta-analytic applications of this method, violations could distort SD_IR_^2^ estimates in predictable ways. If the intervention group exhibits greater within-individual variance than the control group—potentially due to training-induced increases in biological variability or differential adherence patterns—our approach would overestimate true between-individual response heterogeneity (SD_IR_^2^). Conversely, if intervention-related factors somehow reduce within-individual variance (through increased physiological stability or stricter monitoring protocols), true heterogeneity would be underestimated. The direction and magnitude of such bias depend on the extent to which training systematically alters day-to-day biological fluctuations or measurement precision.

Certain populations may be particularly vulnerable to violations of this assumption. Older adults, individuals with chronic diseases, or those undergoing high-intensity training protocols may exhibit different patterns of within-individual variability in response to structured exercise interventions compared to passive control conditions. For instance, frail older adults might experience greater day-to-day fluctuations in physiological readiness during training due to recovery demands, while younger healthy individuals might show more stable within-individual variance across conditions. Similarly, populations with high baseline variability in health status (e.g., metabolic syndrome patients) may demonstrate differential changes in biological fluctuations when exposed to exercise interventions.

Ideally, verification of equal within-individual variance would require primary studies to report data from repeated baseline testing in both groups (e.g., duplicate pre-intervention measurements separated by several days), which would allow for direct calculation and comparison of within-individual variance components. However, this level of detailed data was not available in the studies included in our meta-analysis. Future trials should consider implementing repeated baseline assessments to enable such verification. Additionally, sensitivity analyses could explore how plausible departures from equal variance assumptions affect SD_IR_ estimates—for example, by recalculating SD_IR_ under scenarios where intervention-group within-individual variance is assumed to be 10%, 20%, or 50% higher or lower than control-group variance. Subgroup analyses stratified by population characteristics (age, health status, training intensity) could also help identify contexts where this assumption is most likely violated.

Consequently, our SD_IR_ estimates are contingent upon this unverified assumption, and should be interpreted cautiously in light of this limitation. Despite this limitation, we maintain that using the control group’s variance as an empirical anchor for non-training-related variability represents a methodological strength compared to approaches that do not account for these sources of variation at all. The control group provides the best available estimate of within-individual variance under non-intervention conditions, and our SD_IR_ calculations explicitly adjust for this baseline variability—an adjustment absent in many previous studies claiming substantial inter-individual response differences.

Fourth, as only English-language publications were included, language bias may have influenced the findings, though the direction of such bias is unclear. Fifth, publication bias represents a potential concern, though its likely direction in this research context differs from conventional clinical trials: if the prevailing expectation in the field is that substantial inter-individual differences exist, studies failing to detect such differences may be less likely to be published, potentially leading to overestimation rather than underestimation of true response heterogeneity in the published literature. This consideration suggests our findings may be conservative. Finally, our analysis was intentionally focused on cardiorespiratory and metabolic outcomes, such as VO_2max_ and blood pressure. This focus was necessary due to the prevalence of these measures in the literature, which provided the statistical power for a meta-analysis. This, however, represents a limitation. The effects of exercise are multidimensional, and future research on heterogeneity may find more promising results in other domains. For instance, cognitive domains (executive function, processing speed) and neuromuscular adaptations (strength gains, motor skill acquisition) may exhibit more complex and individualized response patterns. These areas are intricately influenced by factors such as genetic background, neural plasticity, and daily environmental stimuli, and may thus possess a naturally wider spectrum of individual responsiveness. Expanding future investigations into these domains will be crucial for a more holistic understanding of inter-individual differences in the response to exercise.

Future research should employ more rigorous study designs and statistical methods to distinguish observed inter-individual variability from random variation and within-individual variability. Beyond physiological adaptations, research should also systematically investigate inter-individual differences in behavioral responses affecting long-term adherence, enjoyment, and sustainability. Even if physiological adaptations show limited inter-individual variability given adequate stimulus, optimizing exercise modality, intensity, and context to individual preferences and constraints remains essential for translating prescriptions into sustained behavior change and health benefits.

To enhance the reproducibility and transparency of our research, we have made the datasets used in this study (Tables S3–S6) publicly available. We hope these data will serve as a resource for future research, including reanalysis, sensitivity analyses, and methodological improvements.

## 5. Conclusions

This systematic review and meta-analysis found that among studies capable of distinguishing true inter-individual response heterogeneity from random variation and within-individual variability, statistically significant genuine inter-individual differences were not detected in cardiorespiratory fitness, cardiovascular function, or metabolic outcomes following standardized aerobic training. These findings challenge the widespread assumption that individuals exhibit large, stable, biologically determined differences in “trainability” for these fundamental adaptations.

However, our conclusions should be interpreted with appropriate nuance: we do not conclude that inter-individual differences are definitively absent, but rather that there is an absence of compelling evidence for their existence at a magnitude detectable with available methods. It is critical to understand that “no evidence of effect” does not equate to “evidence of no effect.” While we found no compelling evidence to support significant inter-individual differences, this does not prove that such differences do not exist; it merely indicates that they have not yet been detected. Our findings indicate insufficient evidence to justify current claims of substantial individual response heterogeneity, but do not preclude the possibility that future research with enhanced precision may reveal modest differences with clinical significance.

## Figures and Tables

**Table 1:** Statistical Methods for Quantifying Aerobic Exercise Adaptation Heterogeneity.

Statistical Approach	Estimated Source of Variation	Interpretation/Use
TE_M_ (TE_M_ = SD_diff_/$\sqrt{2}$)	Random measurement error (within-subject, test–retest).	Quantifies the “noise” from the measurement tool and day-to-day biological fluctuation.
Linear Mixed Effects Model: Fixed Factor: Training Group Random Factor: Participant Identity	Direct estimate of the subject-by-training interaction (true inter-individual response variance).	Determines each individual’s consistent, repeatable response to the training intervention by isolating it from random and within-subject variation.
SD_IR_ = $\sqrt{{SD}_{EX}^{2}-{SD}_{CTRL}^{2}}$	Inter-individual response variance attributable to exercise (group-based), net of random and within-subject variation assuming equality across groups.	Preferred practical estimator in parallel-arm trials; relies on equal error structure between groups and sufficient sample size.
TE_Δ_ (TE_Δ_ = SD_diff_/$\sqrt{2}$)	Estimate of within-subject variation (from a control group).	Estimates the “noise” (error) expected in change scores when no intervention is applied; used to set response thresholds or calculate confidence intervals.
SD_IR_ = $\sqrt{{SD}_{EX}^{2}-\sqrt{2*CV}}$	Inter-individual response variance after removing random measurement error only; within-subject variation remains.	Approximate/upper-bound when a control group is unavailable; validity depends on transferability of reliability sample and prior trial duration.

TE_M_, typical error of a measurement; SD_diff_, standard deviation of the difference score; SD_IR_, standard deviation of individual responses; TE_Δ_, typical error of a change score.

## Data Availability

No new data were created.

## Supplementary Materials

The following supporting information can be downloaded at: https://www.mdpi.com/article/10.3390/life15121932/s1. Table S1: PPRISMA checklist, Table S2: Search Strategy, Table S3: Study Charactristics01, Table S4: Risk of bias assessment, Table S5: Study Charactiristics02, Table S6: Study Charactiristics03. Additional background information are provided in the Supplementary Materials including references [58,59,60,61,62,63,64,65,66,67,68,69,70,71,72,73,74,75,76,77,78,79,80,81,82,83,84,85,86,87,88,89,90,91,92,93,94,95,96,97,98,99,100,101,102,103,104].

## Appendix A

### Appendix A.1. Standard Deviation of Change Scores

A meta-analysis was conducted on the changes in standard deviation between the exercise group and control group in selected studies to compare the confidence limits of the standard deviation changes between these groups. This equation was used to calculate the standard error of the standard deviation of change scores, which is necessary for the subsequent meta-analysis of variability. For the meta-analysis, the standard error (SE) of the SD estimates was calculated using the following equation [105]:

(A1) $$\sigma (SD)=SD\sqrt{1-\frac{2\lambda^{2}}{n-1}}$$

where **λ** = Γ$\left(\frac{n}{2}\right)$/Γ$(\frac{n-1}{2}$).

### Appendix A.2. Inter-Individual Differences in Adaptation

To isolate the variability specifically due to the exercise intervention, we calculated the SD_IR_. This metric subtracts the variability observed in the control group (representing measurement error and natural fluctuation) from the variability in the exercise group. A resulting SD_IR_ significantly greater than zero suggests true inter-individual differences in training response.

The standard deviation of individual response (SD_IR_) using the following equation [9,10,13,17]:

(A2) $${SD}_{IR}=\sqrt{{SD}_{EX}^{2}-{SD}_{CTRL}^{2}}$$

SD_EX_ and SD_CTRL_ represent the standard deviation change values for the exercise group and control group, respectively. If SD_EX_ > SD_CTRL_, this indicates inter-individual differences in aerobic training adaptation, as the exercise group exhibits additional variability after accounting for measurement error and intra-individual variation (approximated by control group variability). If SD_EX_ ≤ SD_CTRL_, this would indicate an absence of statistical evidence for additional inter-individual differences in adaptation to aerobic training.

The standard error (SE) for each SD_IR_ value was calculated using the following equations to construct 95% confidence intervals (CIs) [9,18]:

(A3) $$SE=\sqrt{2(\frac{{SD}_{EX}^{4}}{n_{EX}-1}+\frac{{SD}_{CTRL}^{4}}{n_{CTRL}-1})}$$

(A4) $$95\%CI=\sqrt{{SD}_{IR}^{2}\pm 1.96\times SE}$$

where $n_{EX}$ and $n_{CTRL}$ represent sample sizes for the exercise and control groups, respectively.

To analyze SD_IR_ values across different studies in the forest plot, SD_IR_ was standardized and ESD_IR_ values were calculated [9,15]. We did not pool studies with substantial heterogeneity in training protocols or participant characteristics in the primary meta-analysis. This approach prioritizes internal validity and accurate effect estimation over broader generalizability.

(A5) $${ESD}_{IR}=\frac{{SD}_{IR}}{{SD}_{baselinePooled}}$$

SD_baselinepooled_ refers to the pooled standard deviation at baseline. If the 95% confidence interval for SD_IR_ is above zero, it indicates inter-individual differences in aerobic training adaptation. If the confidence interval crosses zero or is negative, it suggests no inter-individual differences in aerobic training adaptation.
